# The association between career stress and disordered eating behaviors in university students: a cross-sectional study

**DOI:** 10.1186/s40359-026-04869-9

**Published:** 2026-07-03

**Authors:** Ezgi Toptaş Bıyıklı, Ali Emrah Bıyıklı

**Affiliations:** Department of Nutrition and Dietetics, Faculty of Health Sciences, Alanya Alaaddin Keykubat University, Antalya, Turkey

**Keywords:** Career stress, Eating behavior, University students, Stress, Emotional eating, Young adults, Disordered eating

## Abstract

**Background:**

University students often experience high levels of stress due to uncertainty about their future careers, competitive job markets, and economic instability. Such stress may not only relate to general well-being but also be associated with maladaptive coping behaviors, including emotional or uncontrolled eating. Understanding these associations is crucial for identifying potential correlates of stress-related disordered eating patterns. This study aimed to examine the relationship between career stress and disordered eating behavior among university students, and to evaluate the moderating roles of gender and body mass index (BMI).

**Methods:**

A cross-sectional survey was conducted with 1,294 students (Mean age = 21.2, SD = 2.5; 60.7% female) from a public university in Turkey. Data were collected using demographic questions, the Korean Career Stress Inventory, and the Dutch Eating Behavior Questionnaire.

**Results:**

Female students scored significantly higher than males in both career stress and eating behavior (*p* < 0.05). Career stress was positively correlated with eating behavior and the regression analysis found that career stress explained 8% of the variance in eating behavior (R² = 0.084; β = 0.29; t = 10.82; *p* < 0.001). Moderation analyses indicated that this positive association intensified as BMI increased, and was more pronounced in males.

**Conclusions:**

Career stress was found to be modestly and positively associated with maladaptive eating behaviors among university students. This association intensifies as BMI increases and is more pronounced in males. Longitudinal, multi-method, and multi-center studies are needed to better evaluate the direction of this relationship and its underlying mechanisms.

## Abstract (plain language summary)

Many university students experience stress regarding their future careers. This type of stress is often linked to how individuals cope with emotions and, for some, may be associated with differences in eating habits. When stress reaches very high levels, students may turn to eating as a coping strategy. In this study, over one thousand university students completed surveys on their career stress and eating behaviors. The results showed a modest relationship between career stress and eating behavior, with students scoring higher in career stress also tending to have higher overall eating behavior scores. Female students reported higher levels of career stress, emotional and external eating behaviors compared to male students. However, the association between career stress and eating behavior was stronger for male students and even more intense among students with a higher BMI. These findings suggest that career-related stress may be associated with unhealthy or maladaptive eating behaviors among young adults. Helping students manage stress in healthy ways through counseling, stress management programs, and career guidance could support better eating habits and overall well-being. Since stress is only one of many factors that may influence eating habits, future longitudinal and multi-method studies are needed to better understand this complex relationship.

## Background

Disordered eating is defined as unhealthy, obsessive, or irregular behaviors and attitudes toward food and eating. When these behaviors reach a level that impairs physical health or psychosocial functioning, they can develop into diagnosable eating disorders, such as anorexia nervosa, bulimia nervosa, and binge eating disorder [1].

Based on psychological theories, Van Strien and colleagues (1986) identified three distinct types of eating behavior relevant to disordered eating. The first is emotional eating, which refers to eating in the absence of internal physiological hunger cues and in disregard of satiety signals, as a means to cope with negative emotions. The second, external eating, describes eating in response to food-related stimuli regardless of internal hunger or satiety states. The third type, restrained eating, reflects the degree to which individuals consciously try to restrict their food intake [2]. The presence of emotional, external, and restrained eating behaviors does not indicate an eating disorder in an individual and does not measure clinical pathology. However, the extreme and rigid adoption of these maladaptive eating behaviors falls within the spectrum of disordered eating. Within this spectrum, behaviors such as emotional and external eating are considered potential precursor behaviors for Binge Eating Disorder (BED), while restrained eating is evaluated as a risk factor for threshold-level eating disorders like Anorexia Nervosa [1, 3].

The university period represents a particularly sensitive phase for eating behaviors due to significant lifestyle transitions. Food preferences established during childhood may undergo modifications as parental supervision decreases, creating a context that is conducive to the development of unhealthy eating habits [4]. Although these unhealthy eating habits do not always signify an eating disorder, engaging in behaviors such as skipping meals, dieting, constant preoccupation with body shape, and resorting to overeating to regulate emotions creates a vulnerability. Particularly when used to cope with distress, such maladaptive patterns may significantly increase the risk of developing full-threshold eating disorders over time. Given that unhealthy dietary patterns acquired during this period are associated with long-term health issues, early identification of potential eating disorders is of critical importance [5].

Among the various factors contributing to these maladaptive eating patterns, stress has been identified as a key determinant. Psychological stress is defined as a specific relationship between an individual and their environment, which is perceived as demanding or exceeding available resources and as posing a threat to well-being [6]. Stress is known to serve as a complex correlate of health alterations through behavioral, neural, and endocrine pathways. It relates to health both directly, via autonomic and endocrine processes, and indirectly, by being associated with changes in health-related behaviors [7]. Eating behaviors are an important component of changes in health-related behaviors. The literature provides strong evidence supporting the association between stress and eating behaviors [7–12].

Stress may be associated with either insufficient or excessive food intake, reflecting bidirectional changes in dietary consumption. Acute stress refers to short-term stress responses to immediate demands and is often associated with a reduction in food intake [13]. In contrast, chronic stress involves prolonged or recurrent exposure to stressors, and for many individuals, the typical response is not food avoidance but a tendency to consume energy-dense foods [14]. Eating is frequently used as a coping strategy to provide emotional relief and manage stress. It is estimated that approximately 35–40% of individuals increase their food intake under stressful conditions [15]. Individual differences in stress-related eating responses are often associated with factors such as the intensity of the stressor and personality traits. Chronic life stress has been linked to a preference for high-energy foods that are rich in sugar and fat [16, 17]. Consequently, stress is regarded as a significant correlate of obesity development [17].

These stress-related mechanisms are particularly important during the university years, as this period represents a phase of significant transitions in the lives of young adults. The process of adapting to a new environment and changes in lifestyle is often accompanied by elevated stress levels among students [18, 19]. Additionally, confronting new challenges related to autonomy, identity, career, and one’s societal role may further relate to stress in this population [20]. Beyond these, uncertainty about post-graduation plans, a competitive job market, and concerns about employment can intensify students’ stress even more [21].

The global rise in unemployment has been linked to feelings of hopelessness and anxiety regarding future careers among university students [22]. Psychological pressure arising from employment uncertainty, high performance expectations, and labor market instability is conceptualized as part of career-related stress, which is considered a critical factor associated with students’ psychological well-being [23]. Anxiety, one of the most commonly observed psychological issues during the university period, has been reported to be closely associated with career-related concerns [24]. In addition to psychological outcomes, career stress may relate to behavioral challenges. To cope with stress and anxiety, students often adopt various strategies, among which health-risk behaviors such as insufficient sleep, physical inactivity, alcohol and tobacco use, and unhealthy eating patterns are frequently observed [20, 25]. Specifically, the uncertainty and perceived lack of control regarding career prospects may challenge emotion regulation processes, potentially leading to maladaptive eating behaviors [3].

Theories of stress coping and self-regulation suggest that career-related stress may influence these eating behaviors through different psychological processes [6, 26]. Emotional eating may emerge as a coping response to anxiety, uncertainty, and distress associated with career-related concerns [7, 13, 25]. External eating may develop as heightened cognitive and emotional load under stress weakens self-regulation processes, making individuals more responsive to external food cues rather than internal hunger and satiety signals [14, 26]. Restrained eating, in contrast, may reflect attempts to regain a sense of control in situations characterized by uncertainty by consciously restricting food intake [2]. Taken together, these mechanisms suggest that career-related stress may affect different eating styles through distinct but related psychological processes.

Considering that career-related concerns constitute a chronic source of stress for many university students, the potential relationship between this stress and maladaptive eating behaviors is particularly noteworthy for college populations. Previous research has demonstrated associations between general stress and eating behaviors among university students [7, 11, 26–30]. However, studies specifically investigating the relationship between career-related stress and maladaptive or potentially disordered eating behaviors remain scarce.

Although findings regarding gender differences in career stress vary, the general stress literature consistently suggests that female students are more vulnerable to psychological distress compared to males [11, 21, 27]. Furthermore, research on eating behaviors indicates that women are more prone to emotional and restrained eating patterns as a coping mechanism [20, 31–34]. Therefore, based on these general tendencies, gender is expected to be a distinguishing factor in this study.

In addition to gender, body mass index (BMI) is also closely associated with stress and eating behaviors. Although the relationship between career stress and BMI has not been directly examined in previous studies, research investigating the association between stress and academic stress with BMI has yielded conflicting results [35–37]. While a positive relationship between eating behaviors and BMI has frequently been reported [12, 27, 32, 33, 38], studies indicating a negative association also exist [34, 35, 39].

The present study aims to address this gap by examining the relationship between career-related stress and eating behaviors in university students, while also investigating whether gender and BMI moderate this association. Understanding this relationship is important, as maladaptive eating behaviors constitute significant risk factors for weight gain, obesity, and the development of clinical eating disorders. Given that career concerns represent a significant source of stress for young adults in college, investigating this relationship may not only enhance understanding of how stress relates to health behaviors in this popullation but also inform student support services aimed at managing problematic eating patterns. Based on the literature, we tested several specific hypotheses: First, based on the theoretical considerations regarding stress and eating behaviors, higher career-related stress is expected to be positively associated with maladaptive eating styles, specifically emotional, external, and restrained eating. Second, regarding demographic differences, we hypothesized that female students would report significantly higher levels of career stress and maladaptive eating behaviors compared to male students. Third, we hypothesized that BMI would be a differentiating factor, expecting students with higher BMI to exhibit higher scores in maladaptive eating styles. Fourth, we hypothesized that career stress would account for a statistically significant proportion of the variance in university students’ eating behaviors. Finally, we hypothesized that the relationship between career stress and eating behavior would be significantly moderated by both gender and BMI.

## Methods

### Study design and participants

This study employed a cross-sectional and descriptive design. The target population consisted of undergraduate students enrolled at a public university in Turkey during the 2023–2024 academic year. The sample size was calculated using G-Power (v3.1.3) software. A small effect size (0.1), a significance level of 0.05, and a statistical power of 0.95 were assumed, resulting in a minimum required sample size of 1,073 participants. The study was conducted with 1,294 participants. Eligible participants were 18 years of age or older, had no known health problems, and voluntarily agreed to take part in the study. A convenience sampling method was employed via online platforms. Although participation was voluntary and non-random, all academic faculties of the university were included in the study, and to increase sample diversity, quota targets were determined for each faculty based on the proportional distribution of the university’s student population. The faculties included in the study were: the Faculty of Health Sciences, Faculty of Medicine, Faculty of Dentistry, Faculty of Economics and Administrative Sciences, Faculty of Education, Faculty of Engineering, Faculty of Art, Design and Architecture, Faculty of Sports Sciences, and Faculty of Aviation and Space Sciences. Participants accessed the online survey via a QR code shared through WhatsApp groups, and data collection continued until these quota targets were approximately reached. Data collection took place between May and June 2024.

### Measures

The data were collected using a questionnaire consisting of 3 sections: [1] a personal information form [2], the Korean Career Stress Inventory (KCSI), and [3] the Dutch Eating Behavior Questionnaire (DEBQ). The questionnaire was administered online via Google Forms. It took participants approximately 10 min to complete the survey. Three attention check questions were included in the survey to ensure data quality.

### Personal information form 

To assess general characteristics of the students, a set of demographic questions developed by the researchers was included in the questionnaire. These items covered variables such as age, gender, body weight, height, faculty, academic year, type of residence and perceived income level and employment status.

### Korean career stress inventory (KCSI)

 The Korean Career Stress Inventory (KCSI) was developed by Choi and colleagues (2011) to measure career-related stress among university students. The original version consists of 20 items across four subscales. In the initial validation study, Cronbach’s alpha coefficients for the subscales ranged between 0.83 and 0.89 [40]. The Turkish adaptation was conducted by Özden and Berk (2017). The scale consists of 3 subscales: Career Ambiguity and Lack of Information (reflecting the stress caused by uncertainty about the future, lack of confidence, and difficulties in finding helpful resources or individuals), External Conflict (representing the stress arising from the mismatch between personal career desires and family/environmental expectations), and Employment Pressure (measuring the anxiety about unemployment or not finding a suitable job after graduation). The scale is scored using a 5-point Likert scale: [5] Always [4], Often [3], Sometimes [2], Rarely, and [1] Never. Items are summed to yield an overall total measure of career stress. Total scores range from 20 (minimum) to 100 (maximum), with higher scores indicating greater levels of career stress. In the Turkish adaptation study, the internal consistency coefficient for the total scale was found to be 0.94. For the subscales, Cronbach’s alpha coefficients were 0.94 for Career Ambiguity and Lack of Information, 0.83 for External Conflict, and 0.86 for Employment Pressure [41].

### Dutch eating behavior questionnaire (DEBQ)

 The Dutch Eating Behavior Questionnaire (DEBQ) was developed by Van Strien and colleagues (1986) based on a Dutch sample and consists of 33 items. The DEBQ consists of three subscales: Emotional Eating, Restrained Eating, and External Eating; 10 items among these include the behavior of Restrained Eating, 13 items about Emotional Eating, and 10 items about External Eating. Emotional Eating refers to the tendency to eat in response to emotional fluctuations. Restrained Eating describes the deliberate restriction of food intake due to concerns about weight gain. External Eating reflects eating behavior triggered by external food-related cues. The scale uses a 5-point Likert-type response format ranging from 1 (Never) to 5 (Very Often). Item 31 is reverse-coded; all other items are scored directly. In the original validation study, Cronbach’s alpha coefficients were reported as 0.94 for Emotional Eating, 0.95 for Restrained Eating, and 0.85 for External Eating [2]. The Turkish adaptation was conducted by Bozan and colleagues (2011), maintaining the original 33 items and 3 subscale structure. The internal consistency coefficients (Cronbach’s alpha) for the Turkish version were reported as 0.97 for Emotional Eating, 0.91 for Restrained Eating, and 0.90 for External Eating [42]. In order to use the DEBQ, permission was obtained from the publisher and sole copyright-holder of the questionnaire, Hogrefe Uitgevers BV, Amsterdam, The Netherlands.

### Ethical considerations

This study was conducted in accordance with the principles of the Declaration of Helsinki. Ethical approval was obtained from Alanya Alaaddin Keykubat University Non-Interventional Clinical Research Ethics Committee, dated 16.04.2024, with approval number 2024/05 (04/08). Participants were informed about the purpose of the study, and written informed consent was obtained from each participant. The study was carried out in full compliance with both the Declaration of Helsinki and the principles of research and publication ethics.

### Statistical analysis

Prior to data collection, the study hypotheses were defined, and an analytical plan was established. Participants who did not complete the online survey (*n* = 127) or failed one or more attention check questions (*n* = 11) were excluded from the analyses. Statistical analyses were therefore conducted on data from 1,294 participants. All analyses were performed using SPSS version 25.0 for Windows (SPSS Inc., Chicago, IL, USA). Descriptive statistics (frequencies, percentages, means, and standard deviations) were used to summarize participant characteristics and key study variables. The normality of the data distribution was assessed using the Kolmogorov–Smirnov test. To test the study hypotheses regarding group differences, independent samples t-tests were conducted to compare Career Stress (KCSI) and Eating Behavior (DEBQ) scores based on gender, while one-way Analysis of Variance (ANOVA) was performed to assess variations in these scores across BMI categories. Relationships between the KCSI and DEBQ scales were evaluated using Pearson’s correlation analysis. To examine the association between total career stress and overall eating behavior, simple linear regression analysis was performed. Additionally, multiple linear regression analyses were conducted to determine the extent to which career stress dimensions (Career Ambiguity and Lack of Information, External Conflict and Employment Pressure) explain the variance in specific eating behavior subscales (Emotional, External and Restrained Eating). Prior to the advanced regression analyses, the continuous predictor variable (career stress) was mean-centered to mitigate potential multicollinearity issues when computing the quadratic and interaction terms. To investigate potential non-linear (curvilinear) associations between career stress (KCSI) and eating behavior (DEBQ), a quadratic polynomial regression analysis was performed by introducing a squared term of the mean-centered career stress into the model. Furthermore, moderation analyses were conducted using multiple linear regression models to examine whether the relationship between career stress (KCSI) and eating behavior (DEBQ) was conditional upon gender and BMI. Two separate regression models were estimated. In each model, the career stress variable was mean-centered, and the respective interaction terms (Career Stress x Gender and Career Stress x BMI) were computed and included. For the gender moderation analysis, gender was dummy-coded (1 = female, 0 = male). The presence of a moderation effect was evaluated by examining the statistical significance of the interaction terms. The explanatory power of the models was reported using R^2^ and adjusted R^2^ values. Statistical significance was set at *p* < 0.05.

## Results

**Table 1:** Demographic Characteristics of University Students

Characteristics	*n*	%
Gender		
Female	786	60.7
Male	508	39.3
BMI classification		
Underweight (< 18.5 kg/m²)	127	9.8
Normal weight (18.5–24.9 kg/m²)	877	67.8
Pre-obese (25–29.9 kg/m²)	233	18.0
Obese (≥ 30 kg/m²)	57	4.4
Departments		
Health Sciences, Medicine and Dentistry	339	26.2
Engineering	174	13.4
Economics and Administrative Sciences	163	12.6
Education	259	20.0
Other	359	27.7
Academic year		
1st year	349	27.0
2nd year	406	31.4
3rd year	312	24.1
4th year and above	227	17.5
Type of residence		
Dormitory	869	67.2
Family home	150	11.6
Student house	275	21.3
Perceived income level		
Income less than expenses	481	37.2
Income equal to expenses	652	50.4
Income more than expenses	161	12.4
Employment status		
Working part time	152	11.7
Working full time	69	5.3
Not working	1073	82.9

The demographic characteristics of the participants are presented in Table 1. The mean age of the students was 21.2 ± 2.5 years, with 39.3% identifying as male and 60.7% as female. According to the BMI classification, 9.8% of the participants were underweight and 4.4% were classified as obese.

**Table 2:** Evaluation of Students’ KCSI and DEBQ Total and Subscale Scores by Gender and BMI

	Gender				BMI				
	Female	Male	Total	*p*	Underweight	Normal	Preobese	Obese	*p*
KCSI score	49.1 ± 17.1	46.1 ± 16.6	47.9 ± 16.9	0.002†	51.9 ± 15.7^a^	47.3 ± 16.5^b^	47.5 ± 18.2	49.2 ± 19.8	0.035‡
Career Ambiguity	23.8 ± 9.4	22.7 ± 8.9	23.4 ± 9.2	0.030†	25.4 ± 8.8	23.0 ± 9.0	23.4 ± 9.9	24.0 ± 10.9	0.061‡
External Conflict	8.3 ± 3.5	8.4 ± 3.6	8.3 ± 3.5	0.584†	8.9 ± 3.3	8.2 ± 3.5	8.6 ± 4.0	8.4 ± 3.6	0.168‡
Employment Pressure	16.9 ± 6.0	14.9 ± 5.7	16.1 ± 6.0	< 0.001†	17.6 ± 5.7^a^	16.0 ± 5.9^b^	15.5 ± 6.3^b^	16.7 ± 7.0	0.013‡
DEBQ Score	90.1 ± 21.6	85.1 ± 21.1	88.1 ± 21.5	< 0.001†	77.6 ± 17.9^a^	87.9 ± 21.3^b^	92.9 ± 22.1^c^	96.0 ± 20.9^c^	< 0.001‡
Restrained Eating	24.2 ± 8.8	23.2 ± 8.2	23.8 ± 8.6	0.054†	18.5 ± 7.9^a^	23.7 ± 8.5^b^	26.6 ± 8.1^c^	25.4 ± 8.5^b.c^	< 0.001‡
Emotional Eating	33.8 ± 14.0	31.1 ± 12.5	32.7 ± 13.5	< 0.001†	27.1 ± 11.6^a^	32.7 ± 13.2^b^	35.0 ± 14.2^b^	36.4 ± 15.5^b^	< 0.001‡
External Eating	32.0 ± 7.4	30.7 ± 7.7	31.5 ± 7.6	0.002†	31.9 ± 8.1	31.3 ± 7.5	31.3 ± 7.6	34.1 ± 7.2	0.051‡

*BMI* Body Mass Index, *KCSI* Korean Career Stress Inventory, *DEBQ* Dutch Eating Behavior Questionnaire *Career Ambiguity*: Career Ambiguity and Lack of Information

*p < 0.05, *Data are presented as mean ± standard deviation (SD),†Calculated with independent samples t-test. ‡Calculated by ANOVA test

^*a, b ,c,*^ Values shown with different letters in the same rows where there are more than two groups are statistically different from each other

The evaluation of students’ KCSI and DEBQ total and subscale scores by gender and BMI is shown in Table 2. The total scores of career stress and the DEBQ were found to be significantly higher in female students compared to male students (*p* < 0.05). The subscale scores for Career Ambiguity and Lack Of Information as well as Employment Pressure were also significantly higher among female students (*p* < 0.05). When KCSI total scores were analyzed according to BMI classification, the underweight group had significantly higher KCSI scores than the normal weight group (*p* < 0.05), while no significant differences were found among the other BMI groups. Regarding DEBQ total scores across BMI classifications, scores increased significantly from the underweight group to the obese group (*p* < 0.05), with the exception of the difference between the pre-obese and obese groups, which was not statistically significant.

Restrained and Emotional Eating Behavior scores were significantly lower among underweight students (*p* < 0.05). Although External Eating scores tended to increase with higher BMI, the differences between groups were not statistically significant (*p* > 0.05).

**Table 3:** Correlations Between Total and Subscale Scores of KCSI and DEBQ

	DEBQ total	DEBQ- Restrained	DEBQ-Emotional	DEBQ-External	KCSI total	Career Ambiguity	External Conflict	Employment Pressure
DEBQ total	1							
DEBQ- Restrained	0.584*	1						
DEBQ-Emotional	0.886*	0.298*	1					
DEBQ-External	0.593*	-0.012	0.390*	1				
KCSI total	0.288*	0.147*	0.253*	0.201*	1			
Career Ambiguity	0.273*	0.136*	0.247*	0.180*	0.953*	1		
External Conflict	0.257*	0.163*	0.237*	0.122*	0.783*	0.677	1	
Employment Pressure	0.238*	0.106*	0.190*	0.216*	0.881*	0.742	0.567*	1

Career Ambiguity: Career Ambiguity and Lack of Information. Pearson correlations; *p < 0.001

The correlations between participants’ total and subscale scores on the KCSI and the total and subscale scores of the DEBQ are presented in Table 3. A small but statistically significant positive correlation was found between total career stress scores and total DEBQ scores (*p* < 0.001), as well as with the DEBQ Restrained Eating (*p* < 0.001), Emotional Eating ( *p* < 0.001), and External Eating (*p* < 0.001) subscale scores.

**Table 4:** Simple Linear Regression Analysis of the Relationship Between Career Stress and Eating Behavior

Dependent Variable	Independent Variable	*R*²	F	Β	t	*p*
Eating Behavior	Career Stress	0.084	117.147	0.288	10.823	< 0.001

Dependent variable: Eating Behavior (DEBQ); Independent variable: Career Stress (KCSI) ; *R* = 0.29; Adjusted R² = 0.08; Method: Enter;*p < 0.001*

Simple linear regression analysis examining the association between career stress and eating behavior is presented in Table 4. The analysis revealed that career stress accounted for a statistically significant proportion of the variance in eating behavior (*p* < 0.001), explaining approximately 8% of the variance (*p* < 0.001).

**Table 5:** Results of Multiple Linear Regression Analyses on the Associations between Career Stress and Eating Behavior Subscales

Variables	DEBQ-Emotional Eating			DEBQ-Restrained Eating			DEBQ-External Eating		
	B	β	*p*	B	β	*p*	B	β	*p*
Constant	23.291	-	0.000	20.211	-	0.000	27.046	-	0.000
KCSI-Career Ambiguity	0.238	0.163	< 0.001*	0.049	0.052	0.259	0.045	0.055	0.232
KCSI-External Conflict	0.487	0.129	< 0.001*	0.315	0.131	0.001*	-0.045	-0.021	0.569
KCSI-Employment Pressure	-0.010	-0.004	0.914	-0.010	-0.007	0.872	0.236	0.188	< 0.001*

B = unstandardized regression coefficient; β = standardized regression coefficient.* DEBQ *Dutch Eating Behavior Questionnaire,*KCSI *Korean Career Stress Inventory*.C*areer Ambiguity: Career Ambiguity and Lack of Information. Standard multiple linear regression analyses (enter method) were conducted for each eating behavior subscale. All career stress subdimensions were entered simultaneously as predictors. DEBQ–Emotional Eating model: *R* = 0.27, adjusted R² = 0.07, F(3, 1290) = 32.36,*p < 0.001.*DEBQ–Restrained Eating model: *R* = 0.17, adjusted R² =0.03, F(3, 1290) = 12.25,*p < 0.001.*DEBQ–External Eating model:*R* = 0.22, adjusted R² =0.05, F(3. 1290) = 21.58,*p* < 0.001. *p < 0.05

Multiple linear regression analyses were conducted to examine the extent to which career stress subscales explained the variance in eating behavior subscales (Table 5). The model for Emotional Eating was statistically significant, indicating that career stress subscales explained 7% of the variance in Emotional Eating (*p* < 0.001). In this model, Career Ambiguity and Lack of Information and External Conflict contributed significantly to the explained variance. The model for Restrained Eating was also statistically significant and explained 3% of the variance (*p* < 0.001). In this model, External Conflict was the only subscale that made a significant contribution. For External Eating, the model was statistically significant as well, with career stress subscales explaining 5% of the variance (*p* < 0.001). Employment Pressure was the sole subscale that contributed significantly to the explained variance. Overall, these findings indicate that different dimensions of career stress explain the variance in eating behavior subscales to varying degrees.

Furthermore, to evaluate whether the relationship between career stress and eating behavior follows a non-linear pattern, a quadratic regression analysis was performed. After mean-centering the career stress variable, a squared term was included in the model. The results showed that the quadratic term was not statistically significant (*B* = 0.0018, *SE* = 0.002, *t* = 1.16, *p* = 0.247), indicating that the association follows a linear rather than a curvilinear (U-shaped or inverted U-shaped) pattern. The inclusion of the nonlinear term did not provide a significant improvement in the model’s explanatory power (*R*² = 0.084).

**Table 6:** Moderation Analyses of Gender and BMI on the Relationship between Career Stress and Eating Behavior

	Model 1 (Gender)				Model 2 (BMI)			
	B	SE	t	*p*	B	SE	t	*p*
Intercept	85.927	0.918	93.58	< 0.001	70.145	2.390	29.35	< 0.001
KCSI	0.445	0.055	8.11	< 0.001	-0.512	0.118	-4.36	< 0.001
Moderator	3.867	1.177	3.29	0.001	0.800	0.103	7.77	< 0.001
KCSI × Moderator	-0.143	0.070	-2.05	0.040	0.038	0.005	7.60	< 0.001

Dependent variable:* DEBQ *Dutch Eating Behavior Questionnaire,* KCSI *Korean Career Stress Inventory was mean-centered prior to the analyses. For Model 1, the moderator is Gender (1 = female, 0 = male). For Model 2, the moderator is Body Mass Index (BMI). The interaction term represents Career Stress × Moderator. Model 1 (Gender): R² = 0.094; Adjusted R² = 0.092; F(3,1290) = 44.66;*p < 0.001. *Model 2 (BMI): R² = 0.126; Adjusted R² = 0.124; F(3,1290) = 61.77;*p < 0.001.*

The results of the moderation analyses of gender and BMI on the relationship between career stress and eating behavior are presented in Table 6. A moderation analysis was conducted to determine whether gender moderated the relationship between career stress and eating behavior (Model 1). The interaction term (KCSI × Gender) was statistically significant (*p* = 0.040), indicating that the association between career stress and eating behavior differs between female and male students. Specifically, the negative interaction coefficient indicates that the positive effect of career stress on eating behavior is weaker for females compared to males. Gender also showed a significant main effect, with female students reporting higher total eating behavior scores overall. The overall model was statistically significant and explained approximately 9% of the variance in eating behavior (*R*² = 0.094, adjusted *R*² = 0.092).

In a separate model (Model 2), a moderation analysis was conducted to examine whether BMI moderated this relationship. The interaction between career stress and BMI was also statistically significant (*p* < 0.001). Specifically, the positive association between career stress and eating behavior becomes stronger as BMI increases. The overall model was statistically significant and explained 12.6% of the variance in total eating behavior (*R*² = 0.126, adjusted *R*² = 0.124).

## Discussion

Changing life conditions, increasing individualization, concerns about the future and career-related uncertainties may elevate career stress among university students [21]. Elevated stress can be associated with alterations in both metabolic and behavioral domains, one of which is maladaptive eating behaviors potentially relating to increased vulnerability to disordered eating [7]. In this study, career stress was found to be positively associated with maladaptive eating behaviors. Specifically, while female students reported higher levels of career stress as well as emotional and external eating compared to male students, moderation analysis revealed that the association between career stress and eating behavior was stronger for male students and even more intense among students with a higher BMI. However, the regression model explained only 8% of the variance, indicating limited predictive utility. This suggests that while career stress may contribute to maladaptive eating behaviors, the magnitude of this association is modest. The remaining findings and their context within the existing literature are discussed below.

In this study, the average career stress score of the students was found to be at a moderate level, with a mean of 47.93 ± 16.98. When examining similar studies in the literature, lower average career stress scores have generally been reported [43–45]. For example, in studies conducted with students enrolled in health-related departments, the average career stress scores were reported as 46.8 and 46.5, respectively [43, 45]. In a study involving sports sciences students, the mean score was found to be 43.84 ± 14.92 [44]. In a study examining students from different departments, similar to the sample in our study, the average career stress score was found to be higher than in the present study (49.28 ± 16.48) [41]. These differences in career stress scores may be attributed to the diversity of academic disciplines and the varying employment opportunities available across universities.

Furthermore, individual differences such as gender and body weight may be associated with the perception of career stress. In this study, female students were found to have significantly higher levels of career stress compared to male students (*p* < 0.05). However, the literature presents mixed findings on this issue: while some studies report higher career stress among males [43], others indicate no significant gender differences [44, 45]. In general stress research, however, it is frequently reported that women tend to experience higher stress levels than men [11, 21, 27, 30]. This suggests that the tendency for female students to report higher levels of general stress may also extend to the specific domain of career-related stress.

Regarding body weight, students classified as underweight were found to have significantly higher average career stress scores compared to normal weight students (*p* < 0.05). While the relationship between career stress and BMI has not been widely examined in the literature, a study by Manoharan and colleagues found a significant association between high stress and lower BMI among university students [35]. On the other hand, some studies have reported no significant relationship between BMI and stress [36]. In contrast, a study focusing on medical students reported that academic stress was associated with an increased risk of being overweight or obese [37].

Parallel to the findings on stress, eating behaviors also showed significant demographic variations. In this study, total DEBQ scores as well as the subscale scores for Emotional Eating, Restrained Eating and External Eating were all found to be significantly higher among female students compared to their male counterparts (*p* < 0.05). This finding is consistent with numerous previous studies, which have also reported significantly higher DEBQ scores among females than males [27, 31–34]. However, there are also studies reporting significantly higher Emotional and Restrained Eating scores among males [39], higher External Eating scores in males [38], or no significant gender differences [35].

Previous studies have consistently shown that emotional eating tends to be more prevalent among women [27, 31–34, 38, 46, 47]. Biological differences between females and males may be reflected in emotions and behaviors. The higher maladaptive eating behavior scores observed among women may be attributed to their more frequent use of eating as a coping mechanism in response to stressful or uncertain situations, as well as the influence of gender roles. Women may be more exposed to social media pressures related to thinness and may experience greater fear of gaining weight compared to men.

In this study, total DEBQ scores, as well as Emotional and Restrained Eating subscale scores, were found to be significantly lower among underweight students (*p* < 0.05). This finding is consistent with previous studies reporting that these eating behavior scores are significantly lower in underweight individuals [12, 27, 32, 33, 38]. However, some findings in the literature contradict the results of this study. For instance, Tengilimoglu-Metin and Gumus (2023) reported significantly higher total, Emotional and Restrained Eating scores among underweight individuals [39]. Similarly, a study conducted among university students in Malaysia found that Emotional Eating was significantly associated with lower BMI (*p* < 0.05) [35]. In a study from Japan, a negative association was found between Restrained Eating and BMI [34]. These varying findings in the literature suggest that the relationship between BMI and maladaptive eating behaviors is complex and may be impacted by individual differences and confounding variables.

One of the key findings of this study is the positive and significant relationship between career stress and maladaptive eating behavior outcomes (*p* < 0.05). Although there are no studies directly examining the relationship between career stress and maladaptive eating behavior, numerous studies have shown an association between general stress and maladaptive eating behavior [7, 9–12, 27, 28, 30]. A meta-analysis investigating the relationship between stress and maladaptive eating behavior reported that stress is associated with a decrease in healthy food intake and an increase in unhealthy food consumption [7]. Several studies conducted among university students have also found a relationship between stress and maladaptive eating behavior [7, 11, 27–30]. High levels of stress among university students have been reported to be associated with increased consumption of fast food and snacks, meal skipping, a tendency toward unhealthy food choices and overeating behaviors [11, 21, 30].

Supporting this contextual link, research conducted in the context of academic stress has yielded similar findings, even in the absence of direct studies on career stress. It has been reported that academic stress among students is linked to increased consumption of unhealthy snacks and sugary foods, while being associated with decreased intake of healthy foods such as fruits and vegetables [9, 48–50]. These findings provide a contextual basis for considering career stress as a potential factor associated with maladaptive eating behaviors in university students.

Among these maladaptive eating behaviors, emotional eating is frequently observed as a common response to the stress of university life. Numerous studies have shown that high levels of stress experienced during university years are associated with emotional eating behaviors [10, 12, 26, 27, 35]. During this period, students tend to adopt maladaptive strategies to cope with stress, particularly using food consumption as a coping mechanism. The process of individualization, efforts to adapt to a new social environment and uncertainties about the future are among the key factors that increase stress levels during this stage. In this context, career-related concerns may also be associated with higher stress levels, which in turn negatively impact eating behaviors.

The observed associations between the KCSI and DEBQ subscales suggest that distinct mechanisms may underlie the relationship between career stress and maladaptive eating behaviors. The link between Career Ambiguity, External Conflict, and Emotional Eating aligns with Affect Regulation Theory [3], implying that students might use food to cope with anxiety derived from uncertainty and interpersonal friction. In contrast, the association between External Conflict and Restrained Eating could reflect a compensatory need for control and autonomy [4]. Finally, the relationship between Employment Pressure and External Eating mirrors the Self-Regulation Resource Depletion model, where high cognitive load potentially weakens inhibitory control against external food cues [26].

To further explore this relationship, the moderating roles of gender and BMI on the relationship between career stress and eating behavior were evaluated. Although female students reported significantly higher levels of career stress, as well as emotional and external eating compared to males, the strength of the association between career stress and eating behaviors was found to be significantly greater in male students. This finding contradicts the general trend frequently reported in meta-analyses, which often identify the female gender as a stronger moderator [51, 52]. However, there are also studies indicating that men’s eating behaviors can be more profoundly affected by stress [30, 53]. Since previous studies have not specifically examined ‘career stress’, the current results suggest that the relationship between career stress and eating behavior in men may differ from responses to general stress conditions. The specific burden created by future anxiety and employment pressure may act as a distinct mechanism triggering maladaptive eating behaviors in male students. Similarly, BMI was found to play a moderating role in this relationship. The positive association between career stress and maladaptive eating behaviors was observed to strengthen as BMI increased. This finding supports the results of Caso and colleagues [9], who demonstrated the moderating role of higher BMI in the relationship between academic stress and unhealthy eating among university students. Individuals with a higher BMI may possess a greater susceptibility to stress-induced eating cues, and their use of eating to cope with stress may explain the observed moderation effect [54].

In this study, career stress was found to be a positive correlate of eating behaviors. However, the regression model explained a modest proportion of the variance, indicating limited predictive utility. This suggests that eating behaviors are complex and influenced by multiple factors beyond career stress. Although previous research has not directly examined this specific relationship, other studies have shown that negative emotional states, such as stress and depression can be associated with eating behaviors [55, 56]. Furthermore, stress has been identified as a positive predictor of the adoption of unhealthy eating behaviors among university students [57]. This study contributes to the literature as one of the first to directly examine career stress as a factor associated with eating behaviors, and indicates that students reporting higher levels of career stress also tend to report more problematic eating patterns. These findings suggest that career stress may be associated with maladaptive eating behaviors and may play a role as a modest contributory factor. Examining career stress alongside other psychological and environmental variables may help clarify the boundaries of this relationship and its unique contribution to maladaptive eating behaviors. Furthermore, longitudinal, multi-method, and multi-center studies are needed to elucidate the direction of this relationship and its underlying mechanisms.

### Limitations and strengths

This study has some limitations that should be addressed in future research. First, although participants were drawn from various faculties, a convenience sampling method was used via WhatsApp and QR codes, and the sample was selected from a single public university in Turkey. As this approach is highly likely to yield a self-selected group that systematically differs from non-participants, the faculty quota approach does not fully resolve selection bias or ensure representativeness. Therefore, the external validity as well as the cultural and contextual generalizability of the findings to all university students is limited. In addition, the cross-sectional design of the study does not allow for the assessment of causal relationships between career stress and eating behavior. In this study, the use of self-reported height and weight data can be considered another limitation. As self-reported anthropometric measures may involve measurement error and potential misclassification, this could affect BMI-based group comparisons and may partly explain some of the inconsistencies observed in BMI findings across stress and eating variables. In addition, the use of self-report Likert-type scales for both career stress and eating behaviors for both predictors and outcomes may have introduced common method variance and social desirability effects, especially given that career anxieties and eating behaviors are sensitive topics. This potentially inflates the observed associations and complicates the interpretation of the small effect sizes. Furthermore, female students outnumbered male students in the sample. This imbalance may affect the interpretation of gender differences in career stress and eating behaviors. In addition, cross-group measurement validity was not fully established; specifically, gender and BMI comparisons were conducted without testing the measurement invariance of the Turkish versions of the KCSI and DEBQ across these groups. Therefore, it cannot be assumed that the scales functioned equivalently, and these group comparisons should be interpreted with caution as they might reflect differential item functioning rather than true mean differences. Another limitation of this study is that important psychological and contextual variables that could affect the relationship between career stress and eating behaviors were not comprehensively evaluated. Considering factors such as depressive symptoms, anxiety, chronic negative affect, body image dissatisfaction, socioeconomic hardship beyond a single perceived income variable, food insecurity, dieting history, physical activity, and sleep in future studies could contribute to a more comprehensive understanding of the observed relationships.

Despite these limitations, this study is significant as one of the first to examine career stress as a correlate of maladaptive eating behaviors. To build on these findings, future research is recommended to control for psychological and socioeconomic variables in order to more clearly isolate the specific contribution of career stress to maladaptive eating behaviors. Furthermore, future studies examining this relationship in different cultural and socioeconomic contexts and testing causal associations through experimental designs will allow for a more comprehensive understanding of this association.

## Conclusions

In conclusion, this study demonstrated a statistically significant and positive relationship between career stress and maladaptive eating behaviors among university students. However, this overall association is modest in magnitude and has limited predictive utility, indicating that eating behaviors are influenced by a multidimensional array of factors beyond career stress alone. While gender-based analyses revealed that female students reported significantly higher scores for both career stress and maladaptive eating behaviors, moderation analyses showed that the association between career stress and eating behaviors was significantly stronger in male students. Furthermore, BMI was found to play a moderating role in this relationship, with the association between career stress and eating behavior strengthening as BMI increased.

While causality and generalizability are limited due to the study’s cross-sectional design and single-university setting, the findings suggest that career-related stress factors may be among the components associated with university students’ eating behaviors and, consequently, their overall health status. In this context, incorporating career-related anxieties as a risk component within university counseling services could be considered a potential preventive strategy to support student well-being. Nevertheless, to definitively establish the directionality of this relationship, identify the underlying biological and psychological mechanisms, and fully assess the validity of potential interventions, future research should prioritize longitudinal, multi-method, and multi-center replication studies. Such comprehensive research designs are essential to move beyond current correlational observations toward more robust levels of evidence.

## Data Availability

Data will be made available on request.

## Abbreviations

DEBQ: Dutch Eating Behavior Questionnaire
KCSI: Korean Career Stress Inventory
BMI: Body Mass Index
ANOVA: Analysis Of Variance
